# Around the Hospitals

**Published:** 1894-05-12

**Authors:** 


### Around the Hospitals.

[Notice to the Managers of Institutions.—Special space is now reserved for the insertion of notices of hospital meetings and festivals. The Editor requests that all notices may reach The Hospital Office, 428, Strand, at least one week before the date of each meeting.]

**The Royal Hospital, Richmond.**—Since the issue of the last annual report of the Richmond Hospital, it has acquired the dignity of prefixing the title of Royal to its name by special command of the Queen, a very natural signification of the interest taken in the institution by the Royal Family. Mr. Neville Reid at the annual meeting said he thought the expenditure unnecessary which was devoted to the painting of the proper title on the exterior and on the collecting boxes, but we think the objection was short sighted, as hospital managers have to recognise that it is necessary to keep their institutions as much before the public notice as possible, if they wish to find adequate funds for their support. Mr. Sydney Holland, one of the most energetic and able of hospital chairmen, has shown an example of this principle at Poplar, where the buildings, being in a condition of alteration and extension, do not present a very attractive appearance. He has caused a large placard to be erected in front of the hospital announcing the Queen's visit to the hospital for accidents, so that all passers by must read, and have their attention drawn to the institution. We emphasise this fact, as the public, from want of practical knowledge, often hastily misjudge the wisdom of some forms of expenditure in hospitals. Some time ago it was hoped to have commenced the building of the Princess May ward for children, an isolation ward, together with an administrative building in connection. It has been estimated that the cost of the whole erection would exceed £7,000, and as not quite £4,000 has been collected, the children's ward and isolation ward only are now to be commenced, the expenditure for these being set down at £2,655. Sir Whittaker Ellis, at the annual meeting, stated that he felt sure that had the whole scheme been placed in hand and completed, that the Richmond people would not have allowed the work to be crippled for want of funds. It is to be hoped that his confidence in the charitable feelings of the neighbourhood will be so speedily justified that long ere the initial steps are taken the whole of the required £7,000 will be subscribed. A special feature at this year's annual meeting was a resolution submitted by the in-patients testifying to their gratitude to the medical and nursing staff of the hospital. During the past year 405 in-patients and 3,172 out-patients were admitted. Casualty and dental cases brought the number of persons benefited up to 5,646. The financial condition of the institution is still unsatisfactory, necessitating the withdrawal of capital to meet current expenses. A proposal by Mr. Neville Reid to institute a house-to-house collection is to be carried into effect.

